# Local field potentials for target localization in centromedian deep brain stimulation for epilepsy

**DOI:** 10.3389/fnins.2026.1855048

**Published:** 2026-07-09

**Authors:** Shruti Agashe, Dipali Nemade, Sihyeong Park, Annie Iniya John Benedict Ashok, Matthew Vestal, Derek Southwell, Gerald Grant

**Affiliations:** 1 Department of Neurology, Duke University, Durham, NC, United States; 2 Department of Neurosurgery, Duke University, Durham, NC, United States; 3 Department of Neurology, Orlando Health Neuroscience Institute, Orlando, FL, United States; 4 Department of Neurology, Mayo Clinic, Rochester, MN, United States; 5 Department of Biomedical Engineering, Duke University, Durham, NC, United States; 6 Department of Neurosurgery, Dartmouth Hitchcock Medical Center, Lebanon, NH, United States

**Keywords:** drug resistant epilepsy, intralaminar complex, neuromodulation, neurostimulation, parafascicular

## Abstract

**Objective:**

To evaluate whether local field potential (LFP) spectral profiles can serve as a candidate “spectral fingerprint” for physiological confirmation of centromedian-parafascicular (CM–Pf) targeting during thalamic deep brain stimulation (DBS) for drug resistant epilepsy (DRE).

**Methods:**

This is a retrospective study of 10 patients (20 leads) who underwent CM-DBS implantation for DRE at a single tertiary center. Postoperative CT and preoperative MRI were co-registered, normalized to the Montreal Neurological Institute (MNI) space, and reconstructed using Lead-DBS software to anatomically localize contacts. BrainSense™ Survey recordings were obtained at least 3 weeks post-implant during routine programming. LFP frequency content was analyzed, and prominent peaks were identified and classified into canonical frequency bands (theta, alpha, beta). These spectral profiles were then mapped to MRI-based anatomical localizations, and statistical tests were applied to assess associations between peak patterns, contact localizations and thalamic subregions.

**Results:**

Contacts were distributed as follows: 50 in the CM, 16 in the Parafascicular (Pf), 15 in the Centrolateral, 8 in the Mediodorsal, and 7 in the Ventrolateral (VL) nuclei. Of the 10 representative spectral localizations confined to the CM/CM-Pf region, 8 (80%) displayed a distinct dual-peak spectral profile with peaks in the theta/low alpha (5.5–9 Hz) and high beta (20–30 Hz) bands (mean frequencies: 7.63 Hz and 21.02 Hz, Fisher’s exact test, *p* < 0.001). Single-peak profiles showed no significant association with specific nuclei (*p* = 0.871). Contacts overlapping other thalamic nuclei more frequently exhibited narrow 10–15 Hz peaks (*p* = 0.005) or triple-peak profiles (*p* = 0.02), suggesting mixed structural contributions.

**Conclusion:**

A dual- band candidate spectral pattern consisting of theta/low alpha and high beta peaks was associated with the CM-Pf region in this cohort. This finding provides early evidence supporting the feasibility of incorporating passive LFP recordings as a physiologic marker of target engagement. Future work to prospectively compare bipolar survey-based localization with monopolar recording strategies could enable development of a state-based, physiologically informed spectral atlas to refine CM-Pf targeting in thalamic neuromodulation for DRE.

## 1 Background

Deep brain stimulation (DBS) has emerged as an effective treatment for drug-resistant epilepsy (DRE), receiving FDA approval for stimulation of the anterior nucleus of the thalamus (ANT) in focal epilepsy (Fisher et al., 2010; Fisher, 2023). There is growing interest in targeting the centromedian (CM) nucleus with DBS and responsive neurostimulation (RNS) devices, where studies have demonstrated efficacy across various epilepsy types, including idiopathic generalized epilepsy (IGE), Lennox–Gastaut syndrome (LGS) (Dalic et al., 2021; Agashe et al., 2022; Park et al., 2024; Piper et al., 2022; Cukiert et al., 2020; Yang et al., 2022), and some focal epilepsies (Nanda et al., 2024).

Seizure outcomes following epilepsy DBS are variable, with suboptimal lead placement identified as a major contributing factor (Fisher et al., 2010; Kaufmann et al., 2024; Salanova et al., 2021). Current methods for target localization in epilepsy include indirect selection based off MRI-visible anatomical landmarks and anatomical atlases, direct target visualization with MRI, intra-operative microelectrode recordings, and stimulation testing (Warren et al., 2020). Target localization is particularly challenging, however, due to the small size and anatomical variability of the nuclei. Additionally there are limited MRI sequences that directly depict thalamic nuclei, including CM (Salanova et al., 2021). Following lead delivery, device programming involves selection of the active contacts and stimulation parameters. Unlike DBS programming for movement disorders, where real-time behavioral feedback and relatively well-characterized LFP signals can be used to guide contact selection and stimulation parameters adjustments, epilepsy does not involve symptoms that can be monitored in real time for improvement, and LFP features of epilepsy DBS targets remain, by comparison, relatively undescribed.

While prior studies of seizure outcomes following CM neurostimulation have attempted to define anatomical “sweet spots” for stimulation, results have been heterogeneous and may vary by epilepsy syndrome (Park et al., 2024; Warren et al., 2022). These challenges underscore the urgent need for more reliable, physiologically informed surgical targeting strategies to enhance DBS efficacy and reduce the burden of empirical programming in epilepsy (Lopes et al., 2022). This study seeks to address this gap by exploring utility of passively recorded local field potentials (LFPs) to guide CM targeting.

The Medtronic Percept™ DBS device with BrainSense™ technology is sensing-enabled to allow LFP recordings through either snapshot or chronic modes (Sanger et al., 2024; Yang et al., 2023; Satzer et al., 2023; Weiss et al., 2016). LFPs in the thalamus are extracellularly recorded electrical signals reflecting the summed local and network-level activity of neurons (Buzsaki et al., 2012; Kajikawa and Schroeder, 2011). Using the BrainSense Survey™ feature, a wide range of LFP signals can be captured across contact pairs, such that peak amplitudes on bipolar measurements reflect differences in electrophysiological environment between contacts (Sanger et al., 2024). This technology is increasingly being explored to identify spectral signatures that could serve as electronic seizure diaries, early biomarkers of therapeutic efficacy, or indicators of optimal stimulation intensity based on suppression of specific frequency bands (Yang et al., 2023; Weiss et al., 2016; Wu et al., 2013; Gregg et al., 2021). If awake, ambulatory LFPs are specific to nuclei targeted in epilepsy, this raises the possibility of extending such signatures to other states (e.g., anesthesia), providing a framework for intraoperative and postoperative validation of lead placement.

This exploratory study explores the feasibility of identifying a “spectral fingerprint” a consistent frequency-domain signature associated with thalamic targets in epilepsy. In this study, we perform BrainSense™ Survey recordings from patients who underwent Medtronic DBS lead targeting of CM for epilepsy treatment. Using LFP survey data from 20 implanted electrodes during wakefulness, we assessed whether consistent, area-specific spectral patterns are associated with MRI atlas-defined anatomical localization. This electrophysiological approach with more deliberate focus on spectral signatures rather than isolated frequency peaks, if confirmed in future outcomes-based prospective studies, has the potential to enhance targeting precision with the goal of improving clinical outcomes in DRE.

## 2 Methods

### 2.1 Surgical procedure

CM-DBS implantation was performed at the Duke University Hospital (primary neurosurgeon, D.S.) based on the recommendation of a multidisciplinary epilepsy surgery conference. CM target selection was performed using a combination of indirect visualization methods, with coordinates defined relative to the posterior commissure (PC) at the level of the anterior commissure–posterior commissure (AC–PC) plane, and direct visualization using MP2RAGE sequences. Lead targeting was performed using various delivery platforms, including the Leksell stereotactic frame (Vantage, Elekta), RoSA robot (Zimmer Biomet), and ClearPoint SmartFrame, Implantation was conducted with the patient under general anesthesia; intraoperative microelectrode recordings and stimulation testing were not performed. One patients was implanted with Medtronic 3,389 leads, while others were implanted with Medtronic SenSight™ leads (Table 1). An intraoperative CT was obtained following lead placement to assess targeting error.

**Table 1:** Baseline characteristics.

Subject	Age at implant (years)	Sex	Etiology	Seizure onset zone	Epilepsy syndrome	Lead type
1	18	M	Genetic	Multifocal and generalized	LGS	B33005
2	37	F	Unknown	Multifocal	None	B33005
3	35	M	Genetic	Generalized	IGE	B33005
4	32	F	Genetic	Generalized	IGE	B33005
5	26	M	Structural	Multifocal and generalized	LGS	B33005
6	17	F	Genetic	Multifocal and generalized	LGS	3,389
7	43	M	Unknown	Multifocal	None	B33005
8	57	M	Unknown	Multifocal/diffuse	None	B33005
9	39	F	Structural	Multifocal	None	B33005
10	25	M	Structural	Multifocal	None	B33005

M, male; F, female; LGS, Lennox Gastaut Syndrome; IGE, Idiopathic Generalized Epilepsy, 3,389 refers to the legacy Medtronic DBS lead, B33005 refers to the Medtronic SenSight directional DBS lead.

### 2.2 Study population and data collection

The study was approved under Duke Institutional Review Board (IRB) Pro00115391. Patients were between ages 17–57 years at the time of implantation. All patients had DRE, three were diagnosed with LGS, two with IGE, and five with difficult-to-localize or multifocal seizure onsets (Table 1). LFP recordings were obtained by primary epileptologist (S.A.) during standard clinical device programming visits. BrainSense™ surveys were conducted in all patients at a minimum of 3 weeks following DBS implantation, to mitigate the influence of acute post-implantation effects on LFP recordings.

### 2.3 Lead reconstructions

This retrospective study included ten patients who had undergone DBS implantation with two leads targeting the CM. A post-implantation head CT was obtained at least 2 weeks post implant to minimize errors with brainshift. For each subject, Lead-DBS software version 3.2^1^ was used to co-register preoperative and postoperative imaging data (Horn and Kuhn, 2015; Horn et al., 2019). Postoperative CT scans were coregistered to preoperative MRI using standard Lead-DBS preprocessing workflow. Following registration, the preoperative MRI was warped to the Montreal Neurological Institute (MNI) 2009b Nonlinear Asymmetric standard space using ANTs. To improve the anatomical accuracy of lead localization, brain shift correction was applied, as implemented within Lead-DBS. Final electrode reconstructions were visualized in relation to thalamic nuclei, using the Krauth-Morel atlas for anatomical reference (Krauth et al., 2010). The surgeon’s assessment of final placement of leads with contacts in the CM were also recorded. After completing co-registration, normalization, and reconstruction using lead DBS software, the data were imported into Lead Group for group-level visualization. Contact localization relative to atlas-defined structures was confirmed using two approaches. First, visual inspection of reconstructed leads in Lead-DBS was used to confirm contact positions within specific atlas regions. Nucleus assignments for each contact were categorized as major or minor depending on the extent to which a particular nucleus encompassed the contact. Beyond visualization-based methods, spatial proximity between each contact and the thalamic nucleus was quantified as the shortest distance from the contact center to the nearest nucleus voxel. However, because this method reports the distance to the closest surface voxel, even contacts well within the structure may yield nonzero distances. Therefore, contacts with a center-to-voxel distance of ≤1 mm were classified as spatially proximal to, or embedded within, the nucleus.

### 2.4 LFP measurements and spectral peak identification

BrainSense™ surveys provide a broad spatial sampling of LFP activity across electrode contacts while stimulation is turned off, capturing peak signal magnitude (μVp) as a function of frequency (Hz). Recordings were obtained in a bipolar configuration, sampled at 250 Hz, using all possible electrode contact pairings per hemisphere. Snapshot survey data was collected in an awake, alert state without evidence of seizures during recordings. Each bipolar trace reflects the differential voltage between two contacts, with peak amplitude indicating the degree of neurophysiological dissimilarity between recording sites: larger peak magnitudes suggest greater divergence in underlying LFP signals, likely reflecting sampling from neuroanatomically or functionally distinct regions, whereas smaller or absent peaks suggest similar local field environments. Raw survey data were exported in JavaScript Object Notation (JSON) format for each patient and analyzed offline using MATLAB R2023b (MathWorks, Natick, MA, USA). Given the use of both directional (segmented) and cylindrical (1 × 4) leads across the cohort, only circumferential ring-level bipolar configurations were included to ensure consistency across subjects. For example, BrainSense™ survey of the left lead resulted in the following contact pairs for analysis: 0–1, 0–2, 0–3, 1–2, 1–3, and 2–3. Each channel’s time-domain signal (~20 s snapshot) was transformed into the frequency domain using the Fast Fourier Transform (FFT). The resulting power spectral density was computed in bins of 0.98 Hz, spanning center frequencies from 0 to 96.68 Hz. Based on empirical survey reliability thresholds and known artifacts in low-frequency ranges, signals below the theta band (<5.31 Hz) were considered artifact-prone and excluded from further analysis.

To evaluate whether removal of the aperiodic background altered peak detection, time-domain LFP signals were analyzed for each channel in all patients. Power spectral densities (PSDs) were computed using Welch’s method, and the FOOOF (Fitting Oscillations & One-Over-F) algorithm was applied to separate aperiodic (1/f) components from oscillatory activity (Yang et al., 2023; Buzsaki et al., 2012; Busch et al., 2023; Gerster et al., 2022). For each channel, periodic peaks were extracted and filtered to retain only those exceeding a channel-specific threshold (75th percentile of peak amplitudes), with up to five peaks per channel recorded. Peak frequencies identified after aperiodic correction closely matched those obtained from the raw spectra, typically within 1 Hz, and the overall spectral profiles (single-, dual-, or triple-peak signatures) were preserved, supporting the robustness of peak characterization across methods.

### 2.5 LFP spatial localization methods

Given the recording configuration utilizing contact pairs, inferring the most appropriate monopolar contact representative of the peak LFP presents a methodological challenge. The four physical contacts on a lead do not generate four fully independent monopolar spectral profiles. Rather, the survey provides a set of differential measurements across contact pairs, often showing a graded spectral pattern along the lead. The analytic goal was therefore not to assign one unique spectrum to each physical contact, but to identify the contact region most likely contributing to the maximal spectral feature. Therefore, for each patient, the electrode contact exhibiting the maximal LFP amplitude was identified using two independent methods. The first method mirrored standard clinical interpretation by restricting analysis to the two bipolar contact pairs exhibiting the highest spectral power. The contact common to both high-power pairs (e.g., for contact pairs 8–9 and 9–10, contact 9) was then designated as the site of maximal LFP activity. The second method applied a weighted averaging scheme in which each bipolar pair contributed to the localization of peak activity, with lower weights assigned to pairs with greater inter-contact separation. Weights were assigned inversely proportional to the distance between the contact of interest and each bipolar pair, giving greater influence on closer pairs (Strelow et al., 2022). Briefly, for each candidate contact (c), each bipolar pair (i,j) contributed according to its peak amplitude (A_{ij}) and its inverse distance from the candidate contact. The localization score was calculated as (S_c = \sum w_{c,ij}A_{ij}), where (w_{c,ij}) represents the normalized inverse-distance weight for that bipolar pair. This approach gives greater weight to adjacent pairs and progressively lower weight to more distant pairs accounting for the expectation that larger signal differences arise from greater spatial divergence.

### 2.6 LFP signature analysis

Spectral analysis was initially performed assuming a dominant frequency component localized to a single anatomical structure. However, given the presence of multi-peak spectral profiles (e.g., 1-, 2-, or 3-peak patterns), a structure-specific spectral profile map was conducted consisting of single or multi-peak signatures. Using the localization methods outlined above, 70% of contacts were concordantly identified across methods. When the two approaches were discordant, candidate contacts differed by no more than one contact level and were still located in the nucleus or at the atlas-defined boundaries. Given the inherent spatial overlap of bipolar recordings, these cases were included in the analysis to create a composite profile for the contact pair. This approach allowed for the integration of physiologically relevant frequency characteristics from adjacent contacts within a single region, resulting in 24 representative composite spectral profiles.

### 2.7 Statistical analysis

Statistical analysis was conducted to investigate the relationship between anatomical localization and LFP spectral characteristics, including single peak frequencies and multi peak signatures. For each anatomical structure, mean peak frequencies across contacts were calculated. Frequency data were analyzed both as continuous variables and categorized into canonical frequency bands: theta (5–8 Hz), alpha (8–12 Hz), low beta (12–20 Hz), and high beta (20–30 Hz). These frequency bands served as a conceptual framework for interpretation rather than discrete, mutually exclusive categories; frequencies near band boundaries (e.g., 6–9 Hz) were treated as part of a continuous spectrum encompassing theta/low alpha ranges. Descriptive statistics, including means and standard deviations, were computed for each frequency measure and anatomical localization.

Continuous spectral data were derived using a hybrid localization method (Section 2.5). Continuous data were compared across thalamic regions using nonparametric tests (Kruskal–Wallis, Mann–Whitney U). For categorical data, chi-square goodness-of-fit tests were used to assess whether specific frequency bands were overrepresented within CM/CM-Pf localizations. Pairwise frequency comparisons (e.g., beta vs. alpha, beta vs. theta, alpha vs. theta) were performed using one-proportion Z or binomial tests. For multi-band peaks (dual or triple), Fisher’s Exact tests were applied to determine whether co-occurring frequency bands were preferentially associated with CM/CM-Pf, contacts overlapping multiple nuclei, or adjacent thalamic territories. Given contacts, bipolar recordings, and spectral profiles from the same patient or lead are not fully independent; the small sample size and sparse categorical structure of the data, we did not perform a mixed-effects model, as this would likely be statistically unstable.

## 3 Results

### 3.1 Comprehensive spatial localization of electrode contacts across thalamic nuclei

**Figure 1:**
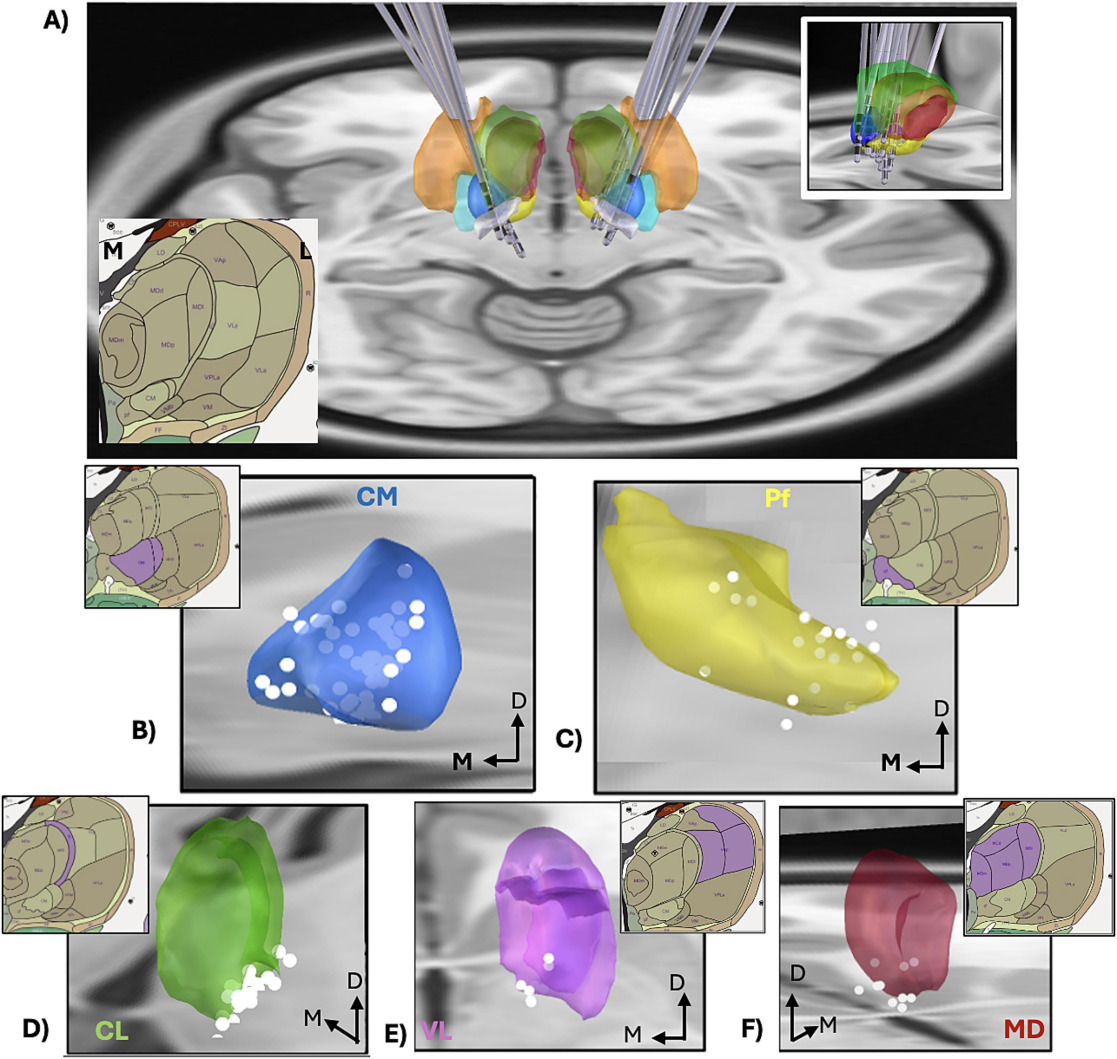
Anatomical localization of 20 reconstructed DBS leads within the centromedian (CM) nucleus and adjacent thalamic nuclei. **(A)** Lead trajectories were reconstructed using the Lead-DBS pipeline and normalized to MNI space. (Top right inset) All 20 leads were mirrored for group visualization. Bottom left inset CM and surrounding nuclei shown in a whole brain atlas. Image courtesy Allen Brain Institute Whole Brain Atlas (https://atlas.brain-map.org/) **(B–F)** Color-coded anatomical segmentation of relevant thalamic nuclei: Blue – Centromedian (CM), Maroon – Mediodorsal (MD), Green – Centrolateral (CL), Yellow – Parafascicular (Pf), Pink – Ventrolateral (VL). Contacts localized within or up to 1 mm from each nucleus are shown as point clouds. Nuclei have been rotated individually for better visualization of point clouds. M, medial; D, dorsal; L, lateral.

Analysis of 80 contacts across 10 subjects revealed the distribution of electrode localizations across multiple thalamic nuclei. Using nearest-voxel–based methods (Section 2.3), the number of contacts localized within or up to 1 mm from each nucleus, including overlapping or nucleus boundaries were as follows: 50 in the CM, 16 in the Pf, 15 in the Centrolateral (CL), 8 in the Mediodorsal (MD), and 7 in the Ventrolateral (VL) nuclei (Figure 1). Fewer than five contacts were localized to the Ventroposteromedial (VPM), Limitans (Li), and Ventromedial (VM) nuclei. This distribution was corroborated by visual-based methods (Sections 2.3 and 2.5), which yielded similar results.

### 3.2 Characterization of single LFP peaks across thalamic contacts

We identified 46 single LFP peaks across all channels. Of the 46 single peaks, 28 mapped to contact regions that were fully or partially localized to the CM, including those overlapping with adjacent thalamic structures. We observed no significant association between single frequency peaks and anatomical structures (Kruskal-Wallis test, *p* = 0.871). Overall, the median frequency of beta peaks in these CM-Pf localized contacts was 20.5 Hz [interquartile range (IQR): 7.32; Q1 = 15.38, Q3 = 22.70]. Across CM-Pf localizations, the chi-square test for single peaks indicated no significant overall difference in the distribution of theta, alpha, and beta peaks (*χ*^2^ = 5.16, df = 2, *p* = 0.076). However, pairwise comparisons revealed that beta peaks were more frequently observed than alpha peaks (83% vs. 17%, *p* = 0.039), while the frequency of beta did not differ significantly from theta (59% vs. 41%, *p* = 0.629). Similarly, theta peaks were more common than alpha, though this difference did not reach statistical significance (78% vs. 22%, *p* = 0.180).

LFPs at frequencies above beta were of lower amplitudes, consistent with the aperiodic structure of neural signals. Spectral widths of the peaks, defined as the number of contiguous 0.98 Hz bins exceeding the mean ± 2 SD threshold of the background spectrum, were compared for peaks above and below 20 Hz. Peaks >20 Hz (*n* = 13) were associated with broader bandwidths than those ≤20 Hz (Mann–Whitney U, z = 2.201, *p* = 0.0139).

### 3.3 Dual-band spectral signatures in the CM-Pf complex

**Figure 2:**
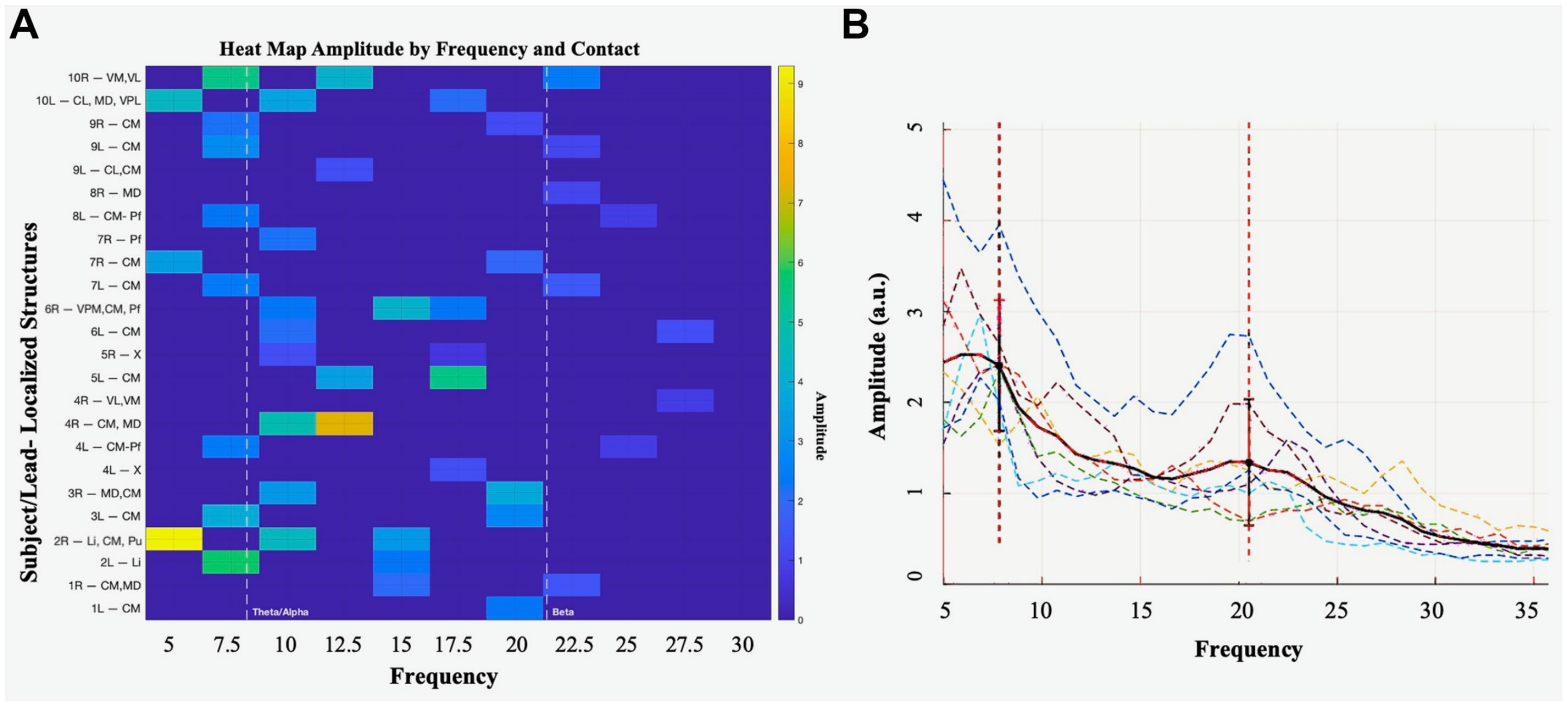
**(A)** Heatmap of amplitude by frequency and contact localization. 24 unique spectral profiles with each row represents anatomical localization of local field potential peaks, labeled by subject number (1–10), laterality (L = left, R = right), and anatomical localization. Centromedian (CM), Mediodorsal (MD), Centrolateral (CL), Parafascicular (Pf), Ventrolateral (VL), Limitans (Li), Ventroposteromedial (VPM), and ventromedial (VM). The X-axis displays frequency (Hz) in 2.5 Hz bins. Profiles are displayed from contact pairs showing highest amplitudes and encompassing the localized contact. Color indicates peak amplitudes (see color bar; warmer colors = higher amplitudes in lower frequencies). Dotted vertical lines demarcate means in dual frequency bands at theta/low alpha and high beta ranges, particularly notable in CM/CM-Pf contacts. **(B)** Raw waveforms from 8 of 10 contact pairs localized exclusively to the CM-Pf region and showing dual peaks, recorded during BrainSense™ Surveys. Waveforms have been vertically shifted so that the highest amplitude is less than 5μVp for visualization purposes; however, spectral flattening was not applied, and signals retain the aperiodic background. (*X* axis 5–40 Hz frequencies in steps of 5 Hz, *Y* axis 1–5 μV peak amplitudes) The solid black line showing peaks in the theta/low alpha and high beta bands represents the mean waveform across all contacts.

**Figure 3:**
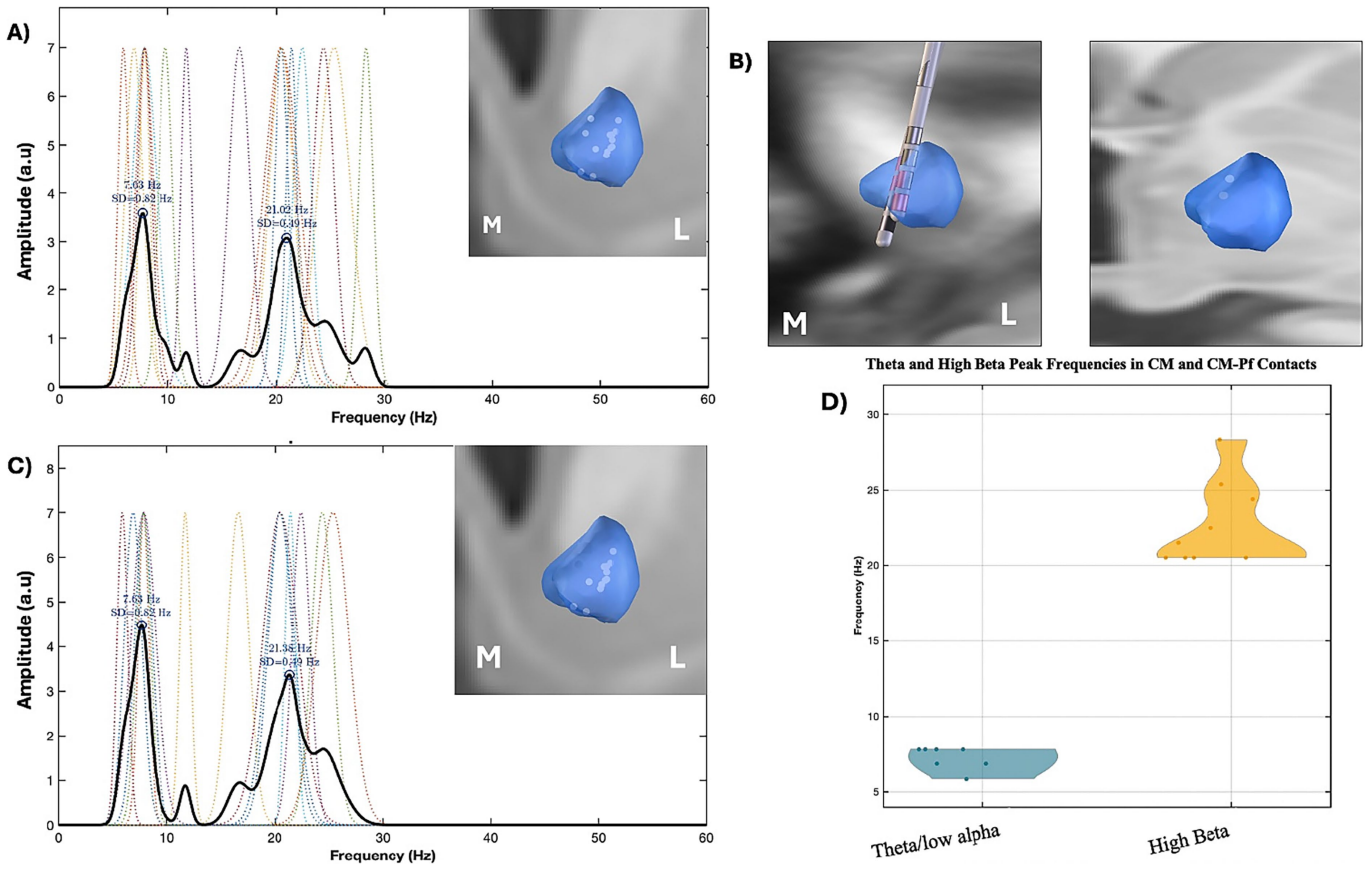
Spectral profiles of LFPs in the CM–Pf complex. **(A)** *Top Left*: spectral power profiles of all LFP recordings from contacts localized exclusively to the centromedian (CM) or CM–parafascicular (CM-Pf) complex (*n* = 10) including bins (spread of frequencies), to highlight spectral distribution independent of amplitude (arbitrary units a.u, not to scale). Inset showing point clouds of ring/segmented contacts in the CM/CM-Pf where LFP peaks were localized. The solid black line denotes the mean spectrum with all CM/CM-Pf localizations. Mean spectral profile shows a dual peaks signature in the low alpha/theta range and high beta range (7.63 Hz, 21.02 Hz respectively). **(B)** *Top Right*: leads whose BrainSense Surveys were without characteristic dual frequency peaks in low alpha/theta and high beta frequencies. Left showing lead trajectories highlighting bipolar contact pairs in CM (red) and right showing 3D point clouds. These contacts were medially placed in the CM. **(C)** *Bottom Left*: plot similar to **(A)** with two leads from **(B)** excluded (*n* = 8/10). Mean spectral profile shows pronounced and smoother separation between the dual peaks in a spectral signature in the low alpha/theta range and high beta range (7.63 Hz, 21.38 Hz respectively) from the CM/CM-Pf. **(D)** *Bottom Right*: violin plots display the distribution of spectral peak frequencies identified within theta/low-alpha (5.5–9 Hz) and high beta (20–30 Hz) bands recorded from thalamic contacts localized exclusively to the centromedian-parafascicular (CM-Pf) complex. Each violin represents the probability density of peak frequencies across contacts, with width proportional to frequency occurrence. M, medial; L, lateral.

**Figure 4:**
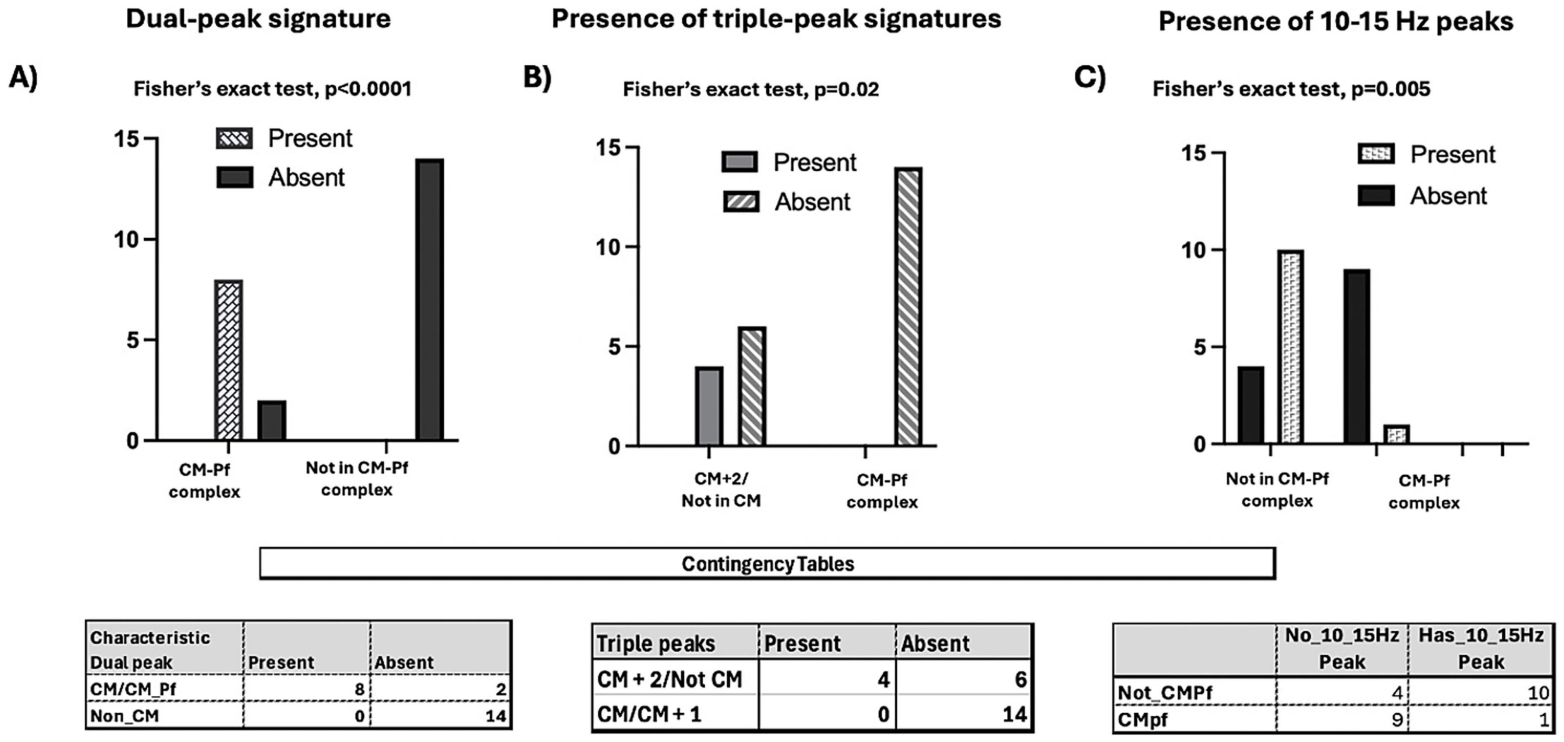
Frequency peak distribution across thalamic nuclei. Top showing stacked bar chart and bottom 2 × 2 contingency table. **(A)** Thalamic contacts in CM/CM–Pf show significantly more dual-band peaks (theta/low alpha 5.5–9 Hz and high beta 20–30 Hz) than other regions (Fisher’s exact test, *p* < 0.001). **(B)** Fisher’s Exact Test (*p* = 0.02) shows triple frequency peaks are significantly associated with contacts overlapping multiple nuclei or outside CM/CM-Pf, **(C)** Contacts overlapping other nuclei (CL, MD, VL, VPL) showed 10–15 Hz narrow peaks, rare in CM/CM–Pf only (Fisher’s Exact Test, *p* = 0.005).

We also assessed the spectral signatures of LFPs that exhibited one or more than one frequency peak. Each thalamic structure was treated as a single unit of analysis, with its spectral signature characterized by single, dual, or triple peaks, resulting in 24 unique profiles (Figures 2, 3). The analysis showed that out of the 10 representative spectral localizations confined to the CM/CM-Pf region without overlap, 8 of 10 (80%) exhibited a distinct dual peak signature with peaks in the theta/low alpha (5.5–9 Hz) and high beta (20–30 Hz) bands, with central frequencies of 7.63 Hz and 21.02 Hz, respectively. This distribution was statistically significant (Fisher’s exact test, *p* < 0.001), supporting early evidence of a candidate dual peak signature within the CM and CM–Pf complex (Figures 3, 4).

Among the 10 representative spectral localizations confined to the CM/CM-Pf region, 2 did not exhibit the characteristic dual-frequency peak in the theta/low alpha and beta ranges. One showed a single beta peak while the other showed peaks in higher alpha and lower beta range. These were both localized medially within the CM (Figure 3B). The remaining profiles with dual peaks were distributed across both medial and lateral portions of the nucleus. Due to the limited number of purely medial contacts, no statistical comparisons were performed.

### 3.4 Spectral signatures associated with non-CM involvement

Spectral profiles solely associated with CM or CM–Pf predominantly displayed a dual-frequency pattern (3c). In contrast, when overlapping other thalamic nuclei, such as the central lateral (CL), mediodorsal (MD), ventral lateral (VL), or ventral posterolateral (VPL), more frequently showed narrow peaks in the 10–15 Hz range. These 10–15 Hz peaks were rare in profiles confined to CM or CM–Pf. Fisher’s Exact Test confirmed a significant association between 10–15 Hz peaks and profiles overlapping these other nuclei (*p* = 0.005) (Figures 2, 4).

There were four spectral signatures consisting of triple peaks. All four triple-peak profiles were associated with either three or more overlapping nuclei (*n* = 3) or two structures located outside the CM/CM-Pf complex (*n* = 1). In contrast, none of the 20 remaining profiles with one or two associated structures confined to the CM/CM-Pf region showed this pattern (Fisher’s Exact Test, *p* = 0.02) revealed a significant association between triple-peak profiles and overlap with multiple nuclei or localization to outside CM/CM-Pf. This provides early evidence supporting this pattern may be associated with recording from structurally heterogeneous or non-CM regions (Figure 4).

## 4 Discussion

Our findings support the presence of a candidate spectral signature within the CM-Pf complex that may serve as a electrophysiological marker to inform CM targeting in epilepsy. When localized to the CM-Pf complex, LFP peaks more frequently exhibited a dual-peak spectral profile, typically comprising a low-frequency component (theta or low alpha range) and a high beta-band oscillation, with peak frequencies of 7.63 Hz and 21.02 Hz, respectively (Figure 3A). This pattern was significantly associated with the CM/CM-Pf complex (Fisher’s exact test, *p* < 0.001), suggesting that this composite oscillatory pattern may be associated within the CM-Pf complex in DRE in an awake, alert state. It is reasonable to hypothesize that the CM–Pf complex, with widespread interactions with cortical, brainstem arousal and basal ganglia regions may give rise to a compound spectral phenotype (Ilyas et al., 2019; Remore et al., 2023). The high beta peak may reflect modulatory input or intrinsic rhythmic activity tied to basal ganglia-thalamocortical loops, while the theta/low alpha component could represent synchronized low-frequency thalamocortical signaling associated with attention, arousal, or sleep–wake transitions.

A study by Satzer et al. (2025) identified thalamic baseline frequencies in patients who had a responsive neurostimulator (RNS) implant. In their study physiological recordings from the CM during periods without interictal activity showed a daytime periodic beta power (12–25 Hz) (Satzer et al., 2025). In our study, all contacts solely localized to the CM/CM-Pf contacts showed a beta oscillation and in 80% we recorded an additional peak at lower frequency (5–9 Hz). Single frequency peaks did not demonstrate statistical separation by structure when analyzed individually (Kruskal–Wallis *p* = 0.871). In a series by Chua et al. (2023), five patients with DRE underwent thalamic DBS targeting either the bilateral ANT (*n* = 3) or CM (*n* = 2). In one CM-implanted patient, initial BrainSense ™ survey recordings identified peaks at 10.74 Hz and 18.55 Hz bilaterally. These findings align with our observation of dual-peak thalamic signatures but differ in the exact frequencies, with our cohort showing relatively lower alpha and higher beta peak centroids. Previous work has shown that the CM exhibits a prominent 10–15 Hz peak under conditions of reduced arousal, such as eye closure or nighttime recordings. We obtained our recordings during wakefulness with eyes open, alert, interactive state without over clinical seizures. None of the spectral profiles localized solely to the CM-Pf complex exhibited a frequency peak in the 10–15 Hz range. Notably, we found that isolated 10–15 Hz activity was more commonly associated with regions outside the CM. (Figure 4, Fisher’s exact test, *p* = 0.005). A spectral spread with triple LFP peaks was observed at anatomical boundaries when localized to multiple thalamic nuclei or had no overlap with the CM-Pf complex (Fisher’s Exact Test, *p* = 0.02).

Prior work in movement disorders has shown that specific brain regions exhibit characteristic oscillatory patterns; for example, the subthalamic nucleus (STN) often displays a beta-band (13–30 Hz) peak, which correlates with symptom severity and therapeutic response in Parkinson’s disease (Fasano et al., 2022; Abu-Hadid and Jimenez-Shahed, 2023; Anwer et al., 2023). In contrast, the use of LFPs in epilepsy remains less explored, and key questions regarding the selection of spectral peaks, their clinical significance, and their relationship to therapeutic efficacy are limited to exploratory studies with most studies focusing on seizure tracking using power-in-band (PIB) data (Yang et al., 2023). Our study aimed to characterize non-ictal awake physiology in DRE and develop spectral signatures to define what constitutes baseline activity in the intended surgical target (CM). LFPs are low-frequency extracellular voltage fluctuations reflecting synchronized activity of local neuronal populations (Yang et al., 2023; Chua et al., 2023; Fasano et al., 2022; van Wijk et al., 2023). While their exact spatial reach remains uncertain, they are generally thought to capture activity within approximately 100–500 microns of the electrode contact, though contributions from more distant structures are likely (Kajikawa and Schroeder, 2011). We assessed structural localization both visually and using voxel-based thresholding within a 1,000 μm radius. While contributions from distal regions cannot be excluded, the consistent presence of a dual-peak spectral profile across contacts suggests this pattern is a feature of the composite signal, irrespective of source distribution.

In this study, we deliberately focused on anatomical targeting rather than an outcome-based “sweet spot” analysis. Outcome-based selection of thalamic stimulation sites is limited due to the delayed nature of feedback (i.e., seizure responses), reliance on retrospective correlations, and the large range of programming parameters (including selection of active contacts, stimulation pathways, programming parameters). As a result, other potentially efficacious contacts are often left unexplored and cannot be definitively ruled out. Variability in CM “sweet spots” across studies likely reflects syndrome-specific differences; the ESTEL trial in Lennox–Gastaut Syndrome favored an anterior inferolateral target, while studies in IGE pointed to a ventromedial CM location (Dalic et al., 2021; Park et al., 2024; Warren et al., 2022). Precise targeting may be fundamental to therapeutic success: it allows delivery of stimulation to the ideal volume of tissue to maximize seizure reduction and simultaneously avoid off-target side effects, and our study addresses this foundational component before linking it to clinical response. Overall, our results suggest that spatial-spectral features of LFPs may correspond to anatomical architecture and potential future studies could guide lead targeting and contact selection.

Prior studies have identified a medial-to-lateral gradient in cytoarchitecture and connectivity of the CM, distinguishing magnocellular from parvocellular subregions (Ilyas et al., 2019; Satzer et al., 2025). In the study using RNS recordings, maximal beta-band activity was localized to the medial CM, suggesting a potential spatial gradient in oscillatory behavior (Satzer et al., 2025). We considered whether subregional differences might contribute to variability in spectral profiles. The two contact pairs showing only a single prominent beta peak or a peak without accompanied 5–9 Hz activity were located medially (Figure 3), whereas those with dual theta/low alpha and high beta peaks were more broadly distributed across the nucleus. Although these preliminary observations suggest a possible link between mediolateral topography and spectral features, the small number of medial-only contacts limits definitive interpretation.

The BrainSense™ survey shows differential activity between two sites rather than the absolute activity at a single contact. The survey often shows a graded spectral pattern along the contacts resulting in 1–2 spectral profiles per lead in our group. The analytic goal was therefore not to assign one unique spectrum to each physical contact, but to identify the contact region most likely contributing to the maximal spectral feature. Given this constraint, inferring the most appropriate contact to represent the peak LFP requires methodological consideration. Therefore, in this study, we applied two independent approaches, the first mirrored clinical practice and the second method used a weighted averaging approach. Recently, the BrainSense Electrode Identifier™ (EI) was introduced to automate contact localization using real-time LFP recordings. EI uses a contralateral lead contact as a reference and when this contact lies in an electrically distinct structure, this configuration may provide a referential derived signal approximating monopolar recording. EI-derived peak localization may offer advantages over manual or weighted methods and future studies should directly compare EI-based localization with the analytic strategies used in our dataset, which relied on BrainSense Survey™ snapshots.

Our ability to characterize spectral patterns beyond the CM was limited by the available sample size and the number of contacts spanning adjacent thalamic regions. While we took steps to optimize anatomical localization, including use of postoperative CT imaging acquired at least 2 weeks after implantation and careful image processing during co-registration and normalization, some degree of uncertainty remains when assigning contacts to small thalamic nuclei. This uncertainty may arise from variability in individual thalamic anatomy, image registration and normalization procedures, and limitations of atlas-based nuclear boundaries in this region. Therefore, the spatial relationship between recording contacts and thalamic nuclei should be interpreted as an estimate of anatomical proximity rather than an absolute assignment. The ≤1 mm criterion was used as a conservative approach to identify contacts most closely associated with atlas-defined regions, but minor localization variability could influence precise nucleus-level attribution of spectral features. Larger cohorts with higher-resolution individual anatomical segmentation will be needed to further validate nucleus-specific spectral signatures. Lastly, we did not perform segment-level analyses due to the heterogeneous use of lead types, including both 1 × 4 and SenSight™ leads. A natural extension of this work would involve systematically characterizing the spectral signatures of neighboring thalamic nuclei. This could serve two key purposes: first, to strengthen anatomical localization by enabling comparisons against a broader spectral reference; and second, to evaluate potential overlap, as non-target nuclei may exhibit similar frequency profiles. This is particularly important given that precise targeting and proximity to neighboring structures is being explored not only to avoid side effects but also to potentially enhance therapeutic outcomes in different epilepsy subtypes (Nanda et al., 2024).

The use of passive electrophysiological recordings from implanted leads to inform thalamic targeting and device programming remains an area of ongoing investigation in neurosurgical practice. Our findings identify an association between anatomical localization within the CM-Pf complex and specific dual-frequency spectral features, suggesting that electrophysiological signatures may provide complementary information to support target characterization. If validated in larger cohorts with clinical outcome correlations, these spectral features could potentially serve as biomarkers to guide contact selection and optimize thalamic neuromodulation strategies. Future studies integrating spectral analyses during implantation, including asleep procedures where thalamic signatures may vary with arousal state, may help evaluate the feasibility of real-time electrophysiological target assessment. Ultimately, development of a comprehensive three-dimensional, state-dependent spectral atlas could provide a framework for improving individualized targeting approaches in thalamic neuromodulation.

## Data Availability

The data analyzed in this study is subject to the following licenses/restrictions: data will be made available upon reasonable request and with data agreements. Requests to access these datasets should be directed to shruti.agashe@duke.edu.

## Footnotes

1
lead-dbs.org
